# The local treatment modalities in FIGO stage I‐II small‐cell carcinoma of the cervix are determined by disease stage and lymph node status

**DOI:** 10.1002/cam4.687

**Published:** 2016-03-14

**Authors:** Juan Zhou, Hong‐Yi Yang, San‐Gang Wu, Zhen‐Yu He, Huan‐Xin Lin, Jia‐Yuan Sun, Qun Li, Zhan‐Wen Guo

**Affiliations:** ^1^ Xiamen Cancer CenterDepartment of Obstetrics and GynecologyThe First Affiliated Hospital of Xiamen UniversityXiamen361003China; ^2^ Xiamen Cancer CenterDepartment of Radiation OncologyThe First Affiliated Hospital of Xiamen UniversityXiamen361003China; ^3^ Department of Radiation OncologySun Yat‐sen University Cancer CenterState Key Laboratory of Oncology in South ChinaCollaborative Innovation Center of Cancer MedicineGuangzhou510060China; ^4^ Department of Radiation OncologyThe General Hospital of Shenyang Military Area CommandShenyang110840China

**Keywords:** Local treatment modalities, radiotherapy, SEER, Small‐cell carcinoma of the cervix, surgery

## Abstract

The purpose of this study was to identify the optimal local treatment modalities for International Federation of Gynecology and Obstetrics (FIGO) stage I‐II small‐cell carcinoma of the cervix (SCCC), including cancer‐directed surgery (CDS) and/or radiotherapy (RT). The Surveillance Epidemiology and End Results (SEER) database was used to identify SCCC patients from 1988 to 2012, and analyzed using Kaplan–Meier survival and Cox regression proportional hazard methods to determine factors significant for cause‐specific survival (CSS) and overall (OS). A total of 208 patients of SCCC were enrolled. The median follow‐up time was 31 months. Fifty‐eight (27.9%) patients were treated with primary CDS, 88 (42.3%) patients underwent CDS combined with RT, and 62 (29.8%) patients were treated with primary RT. Univariate and multivariate analyses showed that local treatment modalities were independent prognostic factors for CSS and OS. Patients who had undergone CDS had better CSS and OS, compared with patients who had been treated with combined CDS and RT or RT alone. The 5‐year CSS and OS of entire group was 49.8% and 46.4%, respectively. The 5‐year CSS in the groups of patients receiving CDS, CDS combined with RT, and RT alone were 67.9%, 49.7%, and 32.6%, respectively (*P *<* *0.001). The 5‐year OS in patients treated with CDS, CDS combined with RT, and RT alone were 64.9%, 46.2%, and 28.8% (*P *<* *0.001). Primary surgery was associated with improved CSS and OS for FIGO stage I and lymph node negative disease. Primary surgery is the most effective local treatment for FIGO stage I‐II SCCC, as adjuvant RT or radical RT does not improve survival compared to radical surgery, especially in patients with FIGO stage I and lymph node negative disease.

## Introduction

Small‐cell carcinoma of the cervix (SCCC) makes up approximately 2–5% of all cervical carcinomas, which shows like highly invasive and distant metastasis 1, 2, 3. The disease‐specific 5‐year survival rates were 36.8%, 9.8%, and 0% for International Federation of Gynecology and Obstetrics (FIGO) stage I–IIA, IIB–IVA, and IVB patients, respectively 4. Currently, most studies on SCCC are comprised of limited series and case reports, making it difficult to draw conclusions on local management in early‐stage SCCC 5.

The Society of Gynecologic Oncology (SGO) and Gynecologic Cancer InterGroup (GCIG) both recommended surgery as the standard local treatment for stage I–IIA SCCC, while chemoradiation was recommended for stage IIB–IV patients 2, 3. However, it has also been suggested that the survival for primary radiotherapy (RT) is superior to primary surgery 6. Given the aggressive nature of SCCC, it is imperative to identify potential treatments that can improve the survival of these patients. Therefore, the aim of the current study was to investigate the optimal local treatment modalities for in stage I–II SCCC using a population‐based national registry (Surveillance Epidemiology and End Results, SEER), which may decrease the potential for selection and surveillance biases typically associated with single‐institution analysis, and provide valuable local treatment modalities for patients with SCCC.

## Patients and Methods

### SCCC case definition

Data were obtained from the current SEER database (Surveillance Research Program, National Cancer Institute SEER*Stat software, Version 8.2.1; http://www.seer.cancer.gov/seerstat), maintained by the National Cancer Institute, which consists of 18 population‐based cancer registries. We obtained permission to access research data files with the reference number 11252‐Nov2014 7. Patients diagnosed with FIGO stage I–II SCCC who had received cancer‐directed surgery (CDS) and/or RT were identified using the SEER database between 1988 and 2012. Pathologic diagnosis was based on the primary site using the International Classification of Disease for Oncology, Third Edition (ICD‐O‐3). SEER database data do not require informed consent, and this study was approved by the ethics committee of the First Affiliated Hospital of Xiamen University.

### Clinicopathological factors

The following clinical and pathologic data were collected from the SEER database: year of diagnosis, age at diagnosis, race, marital status, FIGO stage, grade, tumor size, nodal status, and local treatment modalities including CDS and/or RT. Vital status, duration of follow‐up, and cause of death were also included.

### Statistical analysis

Chi‐squared (*χ* ^2^) and Fisher's Exact probability tests were used to analyze the differences between qualitative data. Univariate and multivariate Cox regression analyses were used to analyze the risk factors for cause‐specific survival (CSS) and overall survival (OS). Multivariable analyses were performed for factors which were significantly associated with CSS and OS in univariate analyses. Calculation of survival rates were plotted by the Kaplan–Meier method, and compared using the log‐rank test. All data were analyzed using SPSS statistical software package, (version 21.0; IBM Corporation, Armonk, NY). A *P*‐value <0.05 was considered to be statistically significant.

## Results

### Patient characteristics and treatment

A total of 208 eligible patients diagnosed with SCCC were identified in the SEER database between 1988 and 2012. Table 1 summarizes the characteristics of the study population. Median age of SCCC diagnosis was 40.5 years (range 22–90 years). There were 144 patients (69.2%) with stage I and 64 patients (30.8%) with stage II SCCC. Of the 150 patients whose histologic grades were available, 147 (98.0%) had poorly differentiated or undifferentiated histology. A total of 58 (27.9%) patients underwent CDS, 88 (42.3%) patients received combined CDS and RT treatment, and 62 (29.8%) patients were treated with primary RT. Of the 62 patients who received primary RT, 24 (38.7%) patients were treated with beam radiation, 33 (53.2%) patients received combination of beam with implants or isotopes, four (6.5%) patients received radioactive implants, and one (1.6%) patient with method or source not specified. Of the 105 patients whose lymph node statuses were available, there were 45 (42.9%) patients with nodal metastases. The local treatment modalities for patients were significantly associated race, FIGO stage, nodal status, and tumor size (*P *<* *0.05).

**Table 1:** Patient characteristics

Variable	*n*	CDS (%)	CDS + RT (%)	RT (%)	*P*
Year of diagnosis					
1988–1992	29	11 (19.0)	9 (10.2)	9 (14.5)	0.104
1993–1997	24	8 (13.7)	14 (15.9)	2 (3.2)	
1998–2002	45	11 (19.0)	20 (22.7)	14 (22.6)	
2003–2007	63	11 (19.0)	28 (31.8)	24 (38.7)	
2008–2012	47	17 (29.3)	17 (19.3)	13 (21.0)	
Age (years)					
<50	140	39 (67.2)	66 (75.0)	35 (56.5)	0.077
≥50	68	19 (32.8)	22 (25.0)	27 (43.5)	
Race					
Black	29	7 (12.1)	6 (6.8)	16 (25.8)	0.015
White	143	39 (67.2)	69 (78.4)	35 (56.5)	
Other	36	12 (20.7)	13 (14.8)	11 (17.7)	
Marital status (*n* = 205)					
Single	96	30 (51.7)	34 (38.6)	32 (54.2)	0.086
Married	109	28 (48.2)	54 (61.4)	27 (45.8)	
Stage					
I	144	47 (81.0)	68 (77.3)	29 (46.8)	<0.001
II	64	11 (19.0)	20 (22.7)	33 (53.2)	
Grade (*n* = 150)					
Well differentiated	1	1 (2.3)	0 (0)	0 (0)	0.392
Moderately differentiated	2	0 (0)	2 (2.9)	0 (0)	
Poorly differentiated or undifferentiated	147	43 (97.7)	67 (97.1)	37 (100)	
Nodal status (*n* = 105)					
Node negative	60	32 (71.1)	28 (46.7)	—	0.012
Node positive	45	13 (28.9)	32 (53.1)	—	
Tumor size (*n* = 138)					
<2 cm	19	13 (32.5)	5 (8.3)	1 (2.6)	0.001
2–4 cm	49	14 (35.0)	25 (41.7)	10 (26.3)	
>4 cm	70	13 (32.5)	30 (50.0)	27 (71.1)	

CDS, cancer‐directed surgery; RT, radiotherapy.

### Analysis of prognostic factors

Univariate analysis showed that age, FIGO stage, nodal status, local treatment modalities were prognostic factors that affected CSS and OS (*P *<* *0.05; Table 2). Race, marital status, and tumor size were not associated with CSS and OS (*P *>* *0.05). Multivariate analysis showed that local treatment modalities were independent prognostic factors for CSS and OS. Patients who received CDS had better CSS than those who had combined CDS and RT treatment (hazard ratio [HR]: 1.831, 95% confidence interval [CI]: 1.057–3.173, *P *=* *0.031) or RT alone (HR: 2.808; 95% CI: 1.590–4.957; *P *<* *0.001). CDS was also associated with improved OS compared to combined CDS and RT (HR: 1.735; 95% CI: 1.038–2.905; *P *=* *0.036) or RT alone (HR: 2.536, 95% CI: 1.486–4.328, *P *=* *0.001). Age was another independent prognostic factor for OS in multivariate analysis (Table 3).

**Table 2:** Univariate analysis of cause‐specific survival and overall survival

	CSS			OS		
Variable	HR	95% CI	*P*	HR	95% CI	*P*
Age (years) (continuous variable)	1.017	1.004–1.029	0.008	1.022	1.010–1.033	<0.001
Race						
Black	1			1		
White	0.662	0.388–1.128	0.130	0.645	0.389–1.070	0.089
Other	0.770	0.400–1.482	0.434	0.761	0.409–1.416	0.389
Marital status						
Single	1			1		
Married	0.923	0.623–1.368	0.690	0.861	0.591–1.254	0.436
Stage						
I	1			1		
II	1.740	1.166–2.597	0.007	1.578	1.071–2.325	0.021
Nodal status						
Node negative	1			1		
Node positive	1.939	1.093–3.440	0.024	1.854	1.065–3.228	0.029
Tumor size (cm)						
<2	1			1		
2–4	1.093	0.516–2.317	0.817	1.067	0.521–2.185	0.858
>4	0.742	0.352–1.564	0.433	0.767	0.378–1.553	0.461
Local treatment modalities						
CDS	1			1		
CDS + RT	1.813	1.044–3.148	0.035	1.725	1.029–2.892	0.039
RT	3.079	1.763–5.377	<0.001	2.906	1.721–4.907	<0.001

CDS, cancer‐directed surgery; RT, radiotherapy; CSS, cause‐specific survival; OS, overall survival.

**Table 3:** Multivariate analyses of cause‐specific survival and overall survival

	CSS			OS		
Variable	HR	95% CI	*P*	HR	95% CI	*P*
Age (years) (continuous variable)	1.012	1.000–1.025	0.059	1.017	1.006–1.030	0.004
Stage						
I	1			1		
II	1.326	0.862–2.040	0.199	1.155	0.763–1.751	0.495
Local treatment modalities						
CDS	1			1		
CDS + RT	1.831	1.057–3.173	0.031	1.735	1.038–2.905	0.036
RT	2.808	1.590–4.957	<0.001	2.536	1.486–4.328	0.001

CDS, cancer‐directed surgery; RT, radiotherapy; CSS, cause‐specific survival; OS, overall survival.

### Impact of local treatment modalities on survival

**Figure 1:**
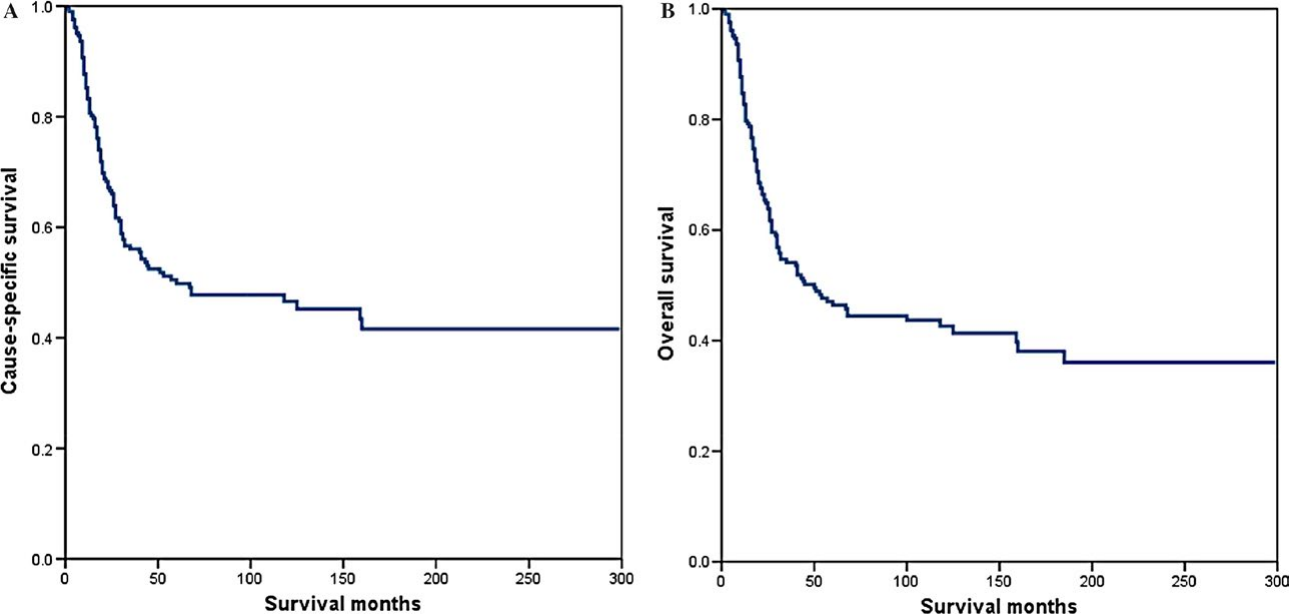
Cause‐specific survival (A) and overall survival (B) of patients with small‐cell carcinoma of the cervix.

**Figure 2:**
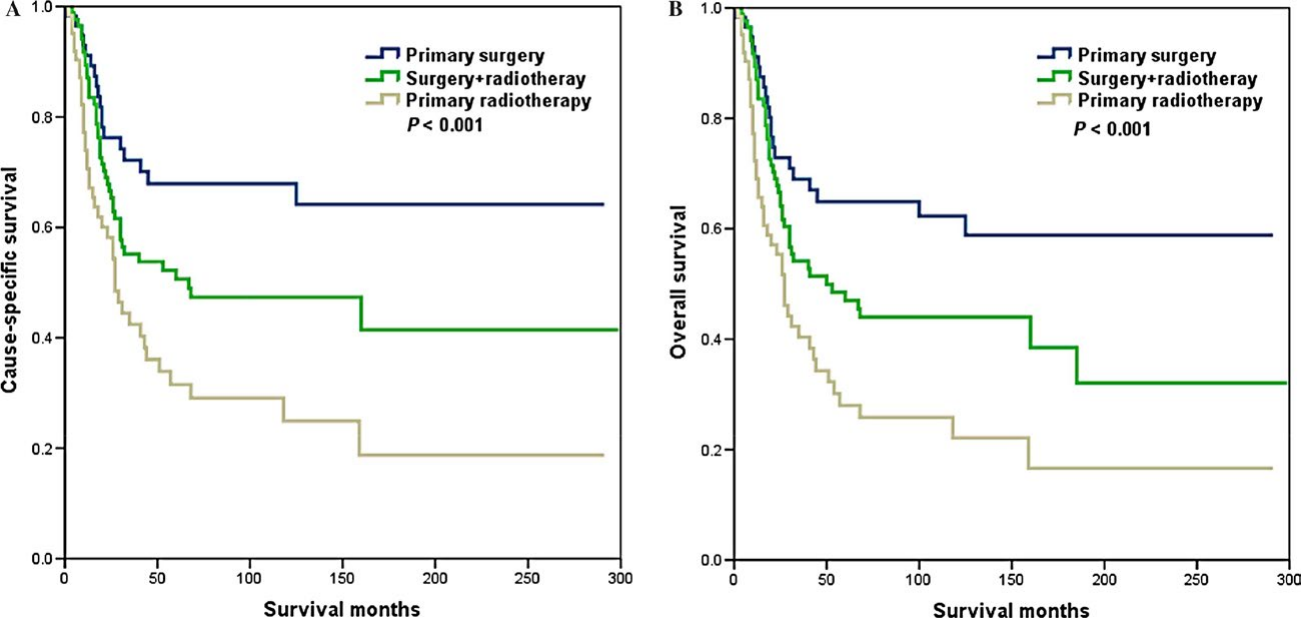
Cause‐specific survival (A) and overall survival (B) of patients with small‐cell carcinoma of the cervix with different local treatment modalities.

The median follow‐up time was 31 months (range, 5–237 months). The 3‐ and 5‐year CSS was 56.0% and 49.8%, respectively (Fig. 1A). The 3‐ and 5‐year OS was 54.1% and 46.4%, respectively (Fig. 1B). The 5‐year CSS according to local treatment modalities of CDS, combined CDS and RT, and RT were 67.9%, 49.7%, and 32.6%, respectively (*P *<* *0.001; Fig. 2A). The 5‐year OS in patients treated with CDS, CDS and RT, and RT alone were 64.9%, 46.2%, and 28.8%, respectively (*P *<* *0.001; Fig. 2B). We further analyzed the effects of radiotherapy methods on the survival of 62 patients who received primary RT, and the results showed that the addition of implants or isotopes did not impact CSS (*P *=* *0.311) and OS (*P *=* *0.228).

**Figure 3:**
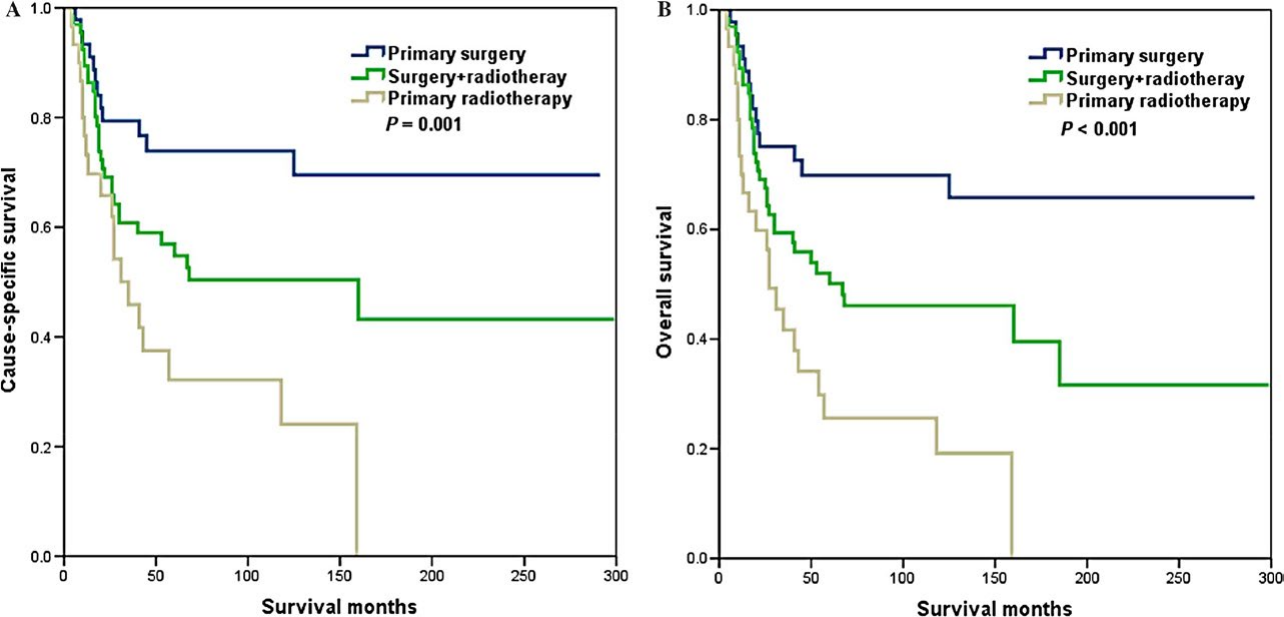
Cause‐specific survival (A) and overall survival (B) of small‐cell carcinoma of the cervix patients with Federation of Gynecology and Obstetrics (FIGO) stage I disease according to different local treatment modalities.

Whether the influence of the local treatment modalities on survival was modified by the FIGO stage was determined. The effect of local treatment modalities significantly differed by FIGO stage; CDS was associated with better CSS (log rank *P *=* *0.001) and OS (log rank *P *<* *0.001) in FIGO stage I SCCC (Fig. 3). However, in FIGO stage II SCCC, no differences were observed in CSS (log rank *P *=* *0.471) and OS (log rank *P *=* *0.557) according to different local treatment modalities.

**Figure 4:**
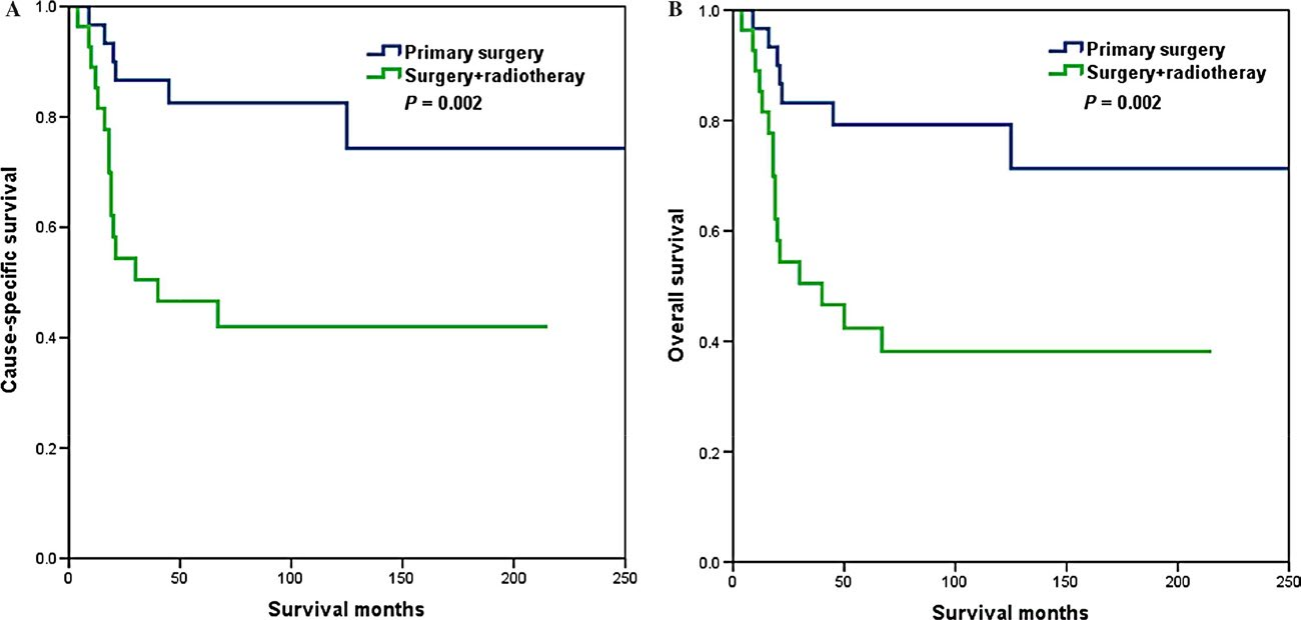
Cause‐specific survival (A) and overall survival (B) of small‐cell carcinoma of the cervix patients with lymph node negative disease according to different local treatment modalities.

The prognostic effects of the different local treatment modalities according to lymph node status were also examined. In patients with lymph node negative disease, local treatment modalities were significantly associated with CSS (log rank *P *=* *0.002) and OS (log rank *P *=* *0.002), and patients who received primary CDS had better survival compared to patients who had combined CDS and RT treatment or RT alone (Fig. 4). In patients with nodal metastases, no associations with local treatment modalities for CSS (log rank *P *=* *0.454) and OS (log rank *P *=* *0.537) were observed.

## Discussion

In this study, we explored the effects of different local treatment modalities on survival of FIGO stage I–II SCCC patients based on SEER data. The results suggested that local treatment modalities are independent factors for CSS and OS in SCCC patients. The survival rate for patients treated with CSD was significantly better than those treated with combined CDS and RT, or RT alone.

Currently, prospective studies have not compared prognosis in SCCC patients with different local treatment strategies, perhaps due to the low prevalence of SCCC. Hoskins et al. described 34 SCCC patients (of which there were 23 patients with stage I–II disease) being treated with combined chemotherapy and radiotherapy, in which 70% of patients achieved complete remission (CR), and the 3‐year OS and failure‐free survival reached up to 60% and 57% 8. A multicenter retrospective study found that for patients with FIGO staged I–II, primary radiotherapy with aggressive chemotherapy was associated with better survival than surgery 6. A study by Viswanathan et al. reported that nine (75%) patients with stage I–II SCCC receiving RT showed disease progression. However, only two patients had disease progression in the radiation field, while the rest of the cases demonstrated extra‐field recurrence or distant metastasis. The study also reported that of the six patients who underwent radical hysterectomy, only two patients (33.3%) showed disease progression in the pelvis and distant organ 9. It has been demonstrated that for patients with a tumor size of ≤2 cm and no vascular invasion, no disease recurrence occurred after treatment with surgery and adjuvant chemotherapy 6, while another study reported that for SCCC patients with stage I–IIA disease, radical hysterectomy could help improve patient survival (38.2% vs. 23.8%; *P *<* *0.001), but RT had no effects on survival (26.9% vs. 36.4%, *P *=* *0.115) 4.

In this study, we have shown that the survival of patients receiving primary CDS was significantly better than patients receiving RT alone or combined with surgery, suggesting that the advantages of local control and radical surgery may be superior to the effects of RT. In line with our findings, both SGO and GCIG also recommend surgical strategies for the treatment of SCCC patients with I–IIA disease, and radio‐chemotherapy for patients with stage IIB–IV disease 2, 3.

SCCC shows highly invasive and distant metastases in its early stages. While studies have shown both local and distant relapses in patients who achieved CR following local treatment, the majority of these relapses occurred in distal locations 10, 11, indicating that distant recurrence, rather than local regional recurrence is the treatment failure for SCCC patients and the value of RT is mainly reflected in the locoregional control. We compared the effects of surgery alone and surgery combined with RT on the survival of patients, and the results showed that the former was significantly superior than the latter. Furthermore, a study by Huang et al. also found that patients receiving RT following surgical treatment showed worse survival than those did who not receive RT 12, while other studies reported no immediate effects on survival for the adjuvant RT after surgery in the treatment of SCCC patients 10, 13. Our study spanned a 25‐year period, during which various modern radiotherapy techniques such as simultaneous integrated boost delivered by intensity‐modulated radiotherapy, or image‐guided brachytherapy were introduced into clinical practice. Therefore, the effect of modern radiotherapy techniques on the survival of SCCC patients need for further study.

In our study, surgery plus RT were mostly applied to the patients with lymph node‐positive and large tumor size. The reason of poor survival for patients received radiotherapy after surgery is not clear; however, it can be assumed that patients receiving adjuvant radiotherapy may have more high‐risk factors of recurrence such as parametrical invasion, lymphovascular invasion, and positive resection margins. Due to the limitations of SEER data, the impacts of lymphovascular invasion, depth of tumor invasion, and surgical margins etc on survival are not clear 14, 15. Hence, we could not determine the reason to receive adjuvant radiotherapy after CDS for SCCC patients.

The use of brachytherapy with whole‐pelvis RT is the standard treatment for cervical cancer. It should be noted that SCCC is a rare disease, and the guidelines for radiotherapy should still refer to cervical cancer. However, in our study, the addition of implants or isotopes impact did not have an impact on CSS and OS in SCCC patients who received primary RT. We could assume that early SCCC was more prone to distant metastases rather than local regional recurrence; the benefit of adjuvant RT or additional brachytherapy was limited to SCCC patients, and the adjuvant chemotherapy in this patient population might be more important 3, 10, 16, 17.

Federation of Gynecology and Obstetrics stage and lymph node status are now considered to be important influence survival factors of SCCC 2, 16, 18, 19. In the present study, subgroup analyses demonstrated that local treatment modalities only significantly influenced CSS and OS in SCCC patients with node negative and FIGO stage I disease. Our results also suggest that surgery primarily benefit patients with a low risk of recurrence, while a more active comprehensive treatment should be explored to improve the patient survival for SCCC patients with a higher recurrence risk.

There are several limitations of this study. The main limitation of the current study is the inherent bias that exists in any given retrospective study. Furthermore, we were unable to accurately evaluate the full extent of the patients' condition based on the insufficient SEER data. SEER database also lacks the information regarding the chemotherapy regimens and dose, which will impact the assessment of the clinical value of local treatment modalities. However, the primary strength of this study is the ability to retrospectively describe the epidemiology, prognostic factors, and treatment trends of this rare disease, using the SEER registry. In addition, there is little information to guide analysis of why postoperative RT was or was not completed in certain SCCC patients. Although retrospective reviews should not carry the power of prospective studies, no prospective assessment of postoperative radiotherapy has been completed in SCCC.

In conclusion, SCCC is a rare type of cervical cancer with highly aggressive feature. There is an urgent need to develop new and better treatments for SCCC. Radical surgery is the optimal local treatment for early‐stage SCCC, as adjuvant or radical RT does not improve survival compare to radical surgery, especially in patients with FIGO stage I and lymph node‐negative disease. Prospective studies are required to confirm the results of this study and help define optimal local management in SCCC.

## Conflict of Interest

None of the authors report any competing interests.

## References

[1] Albores‐Saavedra, J. , D. Gersell , C. B. Gilks , D. E. Henson , G. Lindberg , L. H. Santiago , et al. 1997 Terminology of endocrine tumors of the uterine cervix: results of a workshop sponsored by the College of American Pathologists and the National Cancer Institute. Arch. Pathol. Lab. Med. 121:34–39. 9111090

[2] Satoh, T. , Y. Takei , I. Treilleux , M. Devouassoux‐Shisheboran , J. Ledermann , A. N. Viswanathan , et al. 2014 Gynecologic Cancer InterGroup (GCIG) consensus review for small cell carcinoma of the cervix. Int. J. Gynecol. Cancer 24:S102–S108. 25341572 10.1097/IGC.0000000000000262

[3] Gardner, G. J. , D. Reidy‐Lagunes , and P. A. Gehrig . 2011 Neuroendocrine tumors of the gynecologic tract: A Society of Gynecologic Oncology (SGO) clinical document. Gynecol. Oncol. 122:190–198. 21621706 10.1016/j.ygyno.2011.04.011

[4] Cohen, J. G. , D. S. Kapp , J. Y. Shin , R. Urban , A. E. Sherman , L. M. Chen , et al. 2010 Small cell carcinoma of the cervix: treatment and survival outcomes of 188 patients. Am. J. Obstet. Gynecol. 203:347e1–347e6. 20579961 10.1016/j.ajog.2010.04.019

[5] Crowder, S. , and E. Tuller . 2007 Small cell carcinoma of the female genital tract. Semin. Oncol. 34:57–63. 17270667 10.1053/j.seminoncol.2006.10.028

[6] Chen, T. C. , H. J. Huang , T. Y. Wang , L. Y. Yang , C. H. Chen , Y. M. Cheng , et al. 2015 Primary surgery versus primary radiation therapy for FIGO stages I‐II small cell carcinoma of the uterine cervix: Aretrospective Taiwanese Gynecologic Oncology Group study. Gynecol. Oncol. 137:468–473. 25797082 10.1016/j.ygyno.2015.03.015

[7] Surveillance, Epidemiology, and End Results (SEER) . Program (www.seer.cancer.gov) SEER*Stat Database: Incidence ‐ SEER 18 Regs Research Data + Hurricane Katrina Impacted Louisiana Cases, Nov 2014 Sub (1973‐2012 varying) ‐ Linked To County Attributes ‐ Total U.S., 1969‐2013 Counties, National Cancer Institute, DCCPS, Surveillance Research Program, Surveillance Systems Branch, released April 2015, based on the November 2014 submission.

[8] Hoskins, P. J. , K. D. Swenerton , J. A. Pike , P. Lim , C. Aquino‐Parsons , F. Wong , et al. 2003 Small‐cell carcinoma of the cervix: fourteen years of experience at a single institution using a combined‐modalityregimen of involved‐field irradiation and platinum‐based combination chemotherapy. J. Clin. Oncol. 21:3495–3501. 12972526 10.1200/JCO.2003.01.501

[9] Viswanathan, A. N. , M. T. Deavers , A. Jhingran , P. T. Ramirez , C. Levenback , and P. J. Eifel . 2004 Small cell neuroendocrine carcinoma of the cervix: outcome and patterns of recurrence. Gynecol. Oncol. 93:27–33. 15047210 10.1016/j.ygyno.2003.12.027

[10] Kuji, S. , Y. Hirashima , H. Nakayama , S. Nishio , T. Otsuki , Y. Nagamitsu , et al. 2013 Diagnosis, clinicopathologic features, treatment, and prognosis of small cell carcinoma of the uterine cervix;Kansai Clinical Oncology Group/Intergroup study in Japan. Gynecol. Oncol. 129:522–527. 23480872 10.1016/j.ygyno.2013.02.025

[11] Zivanovic, O. , M. M jr. Leitao , K. J. Park H. Zhao J. P. Diaz , J. Konner , et al. 2009 Small cell neuroendocrine carcinoma of the cervix: analysis of outcome, recurrence pattern and the impact ofplatinum‐based combination chemotherapy. Gynecol. Oncol. 112:590–593. 19110302 10.1016/j.ygyno.2008.11.010

[12] Huang, L. , L. M. Liao , A. W. Liu , J. B. Wu , X. L. Cheng , J. X. Lin , et al. 2014 Analysis of the impact of platinum‐based combination chemotherapy in small cell cervical carcinoma: amulticenter retrospective study in Chinese patients. BMC Cancer 14:140. 24575810 10.1186/1471-2407-14-140 PMC3939817

[13] Intaraphet, S. , N. Kasatpibal , S. Siriaunkgul , A. Chandacham , K. Sukpan , and J. Patumanond . 2014 Prognostic factors for small cell neuroendocrine carcinoma of the uterine cervix: an institutional experience. Int. J. Gynecol. Cancer 24:272–279. 24401981 10.1097/IGC.0000000000000059

[14] Kasamatsu, T. , Y. Sasajima , T. Onda , M. Sawada , T. Kato , and M. Tanikawa . 2007 Surgical treatment for neuroendocrine carcinoma of the uterine cervix. Int. J. Gynaecol. Obstet. 99:225–228. 17897648 10.1016/j.ijgo.2007.06.051

[15] Chan, J.K. , V. Loizzi , R. A. Burger , J. Rutgers , and B. J. Monk . Prognostic factors in neuroendocrine small cell cervical carcinoma: a multivariate analysis. Cancer 97:568–574. 12548598 10.1002/cncr.11086

[16] Lee, J. M. , K. B. Lee , J. H. Nam , S. Y. Ryu , D. S. Bae , J. T. Park , et al. 2008 Prognostic factors in FIGO stage IB‐IIA small cell neuroendocrine carcinoma of the uterine cervix treatedsurgically: results of a multi‐center retrospective Korean study. Ann. Oncol. 19:321–326. 17962205 10.1093/annonc/mdm465

[17] McCann, G.A. , C. E. Boutsicaris , M. M. Preston , F. J. Backes , E. L. Eisenhauer , and J. M. Fowler , et al. 2013 Neuroendocrine carcinoma of the uterine cervix: the role of multimodality therapy in early‐stage disease. Gynecol. Oncol. 12:135–139. 23357610 10.1016/j.ygyno.2013.01.014

[18] Intaraphet, S. , N. Kasatpibal , M. Søgaard , S. Khunamornpong , J. Patumanond , A. Chandacham , et al. 2014 Histological type‐specific prognostic factors of cervical small cell neuroendocrine carcinoma, adenocarcinoma, and squamous cell carcinoma. Onco. Targets Ther. 7:1205–1214. 25061322 10.2147/OTT.S64714 PMC4085311

[19] Liao, L. M. , X. Zhang , Y. F. Ren , X. Y. Sun , N. Di , N. Zhou , et al. 2012 Chromogranin A (CgA) as poor prognostic factor in patients with small cell carcinoma of the cervix: results of aretrospective study of 293 patients. PLoS ONE 7:e33674. 22529895 10.1371/journal.pone.0033674 PMC3328482

